# Positive psychology interventions during pregnancy: A systematic review

**DOI:** 10.1111/aphw.70206

**Published:** 2026-08-28

**Authors:** Tharunnia M.S Ganesan, Zuhrah Beevi, Alan J. Gow, Guek Nee Ke

**Affiliations:** ^1^ Department of Psychology Heriot‐Watt University Putrajaya Malaysia; ^2^ Smart Mind Psychotherapy Solutions Selangor Malaysia; ^3^ Department of Psychology Heriot‐Watt University Edinburgh UK

**Keywords:** maternal health, positive psychology interventions, pregnancy, pregnant women, well‐being

## Abstract

Positive psychology interventions (PPI) have been applied and demonstrated evidence in various population groups. The present systematic review focused on the types and influence of PPI on the physical and psychological health of pregnant women. Studies that matched the selection criteria were identified on EBSCOhost, PsychINFO, Web of Science, PubMed, Scopus and four positive psychology journals. From the 2528 records identified, finally eight studies were included in the review. PPI in this review were delivered utilising various positive psychology components such as hope, gratitude and optimism based on existing theories, for example, the strengths theory, broaden‐and‐build theory, and hope theory. Most interventions were conducted from 14 gestational weeks onwards and were delivered via virtual platforms or mobile applications. As a result of this systematic review, it was identified that PPIs for maternal well‐being were aimed at improving (1) physical health, including labour pain, nausea and vomiting; (2) psychological health, including stress, emotions, anxiety and depression; and (3) subjective health, including life satisfaction, perceived social support and quality of life. Most of the selected studies provided significant evidence towards improvement of well‐being outcomes from administering PPI. For future studies, in‐depth PPI integrated coping and support approaches should be further evidenced among diverse pregnant populations.

## INTRODUCTION

Pregnancy is a transitional time for women as they learn about and adapt to physical, psychological and emotional changes. As such, past studies (Carmona‐Monge et al., 2012; Guardino & Schetter, 2014; Ibrahim et al., 2019) have focused on the importance of coping and support during pregnancy towards the maintenance of positive well‐being. One such coping method and strategy has stemmed from the field of positive psychology (Folkman & Moskowitz, 2003). Positive psychology was first defined by Seligman and Csikszentmihalyi (2000) based on three levels: subjective, individual and group. Interpersonal skills, courage and forgiveness relate to positive psychology on an individual level, whereas altruism, nurturance and tolerance are examples of group level experiences. As an extension, positive psychology interventions (PPI) as a coping and support resource have been applied in healthy adults (Antoine et al., 2018), older people (Cantarella et al., 2017), women with breast cancer (Casellas‐Grau et al., 2013), patients with major depression (Carr & Finnegan, 2015), university students (Guo et al., 2016) and women with fertility challenges (Asl et al., 2016). Corno et al. (2019) produced a narrative review on PPIs during the perinatal period, focussing on mental health, whereas Guardino and Schetter (2014) conducted a systematic review that specifically considered coping, one aspect of PPI, during pregnancy. To the best of our knowledge, however, there has not been a systematic consideration of positive psychology interventions for pregnant women encompassing overall physical and psychological well‐being outcomes. We therefore used a systematic review approach to explore the benefits of PPI and their application specifically for the improvement of well‐being in pregnant women.

PPI have been defined as any intervention that increases overall positive feelings, behaviours and cognitions (Sin & Lyubomirsky, 2009). Others, however, have suggested that PPI are interventions that utilise strategies anchored on specific existing positive psychology theories and empirical evidence that would increase one's well‐being (Hendriks et al., 2019). In a broader adoption of the PPI definition utilised in Carr and colleague's work (2023), Schueller et al. (2014) highlight the need for these interventions to have a ‘goal’ towards improvement of well‐being, and this is then achieved through ‘pathways’ within or beyond contemporary positive psychology models. Examples of PPI included were yoga, goal setting exercises, savouring of past and present memories, optimism and reinforcing positive characteristics towards oneself (Carr et al., 2023). In the context of a systematic review, positive psychology interventions were defined as increasing overall positive feelings, behaviours and cognitions on top of utilising existing theories and strategies that would increase one's well‐being (Hendriks et al., 2019). Specific applications of these components were used as an objective in a more recent systematic review of PPI that investigated how PPI increased well‐being, quality of life and its influence on psychological symptoms such as depression, stress and anxiety (Carr et al., 2021). In addressing the pathway and end goal of these interventions, related measures and scales used within PPI have been utilised to test a range of outcome measures in particular subjective and psychological well‐being (Bolier et al., 2013), depression (Celano et al., 2017), stress (Cullen et al., 2016), anxiety (Hendriks, 2018) and quality of life (Koydemir & Sun‐Selisik, 2016).

### Positive psychology during pregnancy

For some women, the experience of varied challenges during the course of pregnancy could result in negative well‐being impacts. Besides managing these impacts via medications and medical interventions, nonpharmacological methods could complement as effective ways of coping and managing health concerns similar to anxiety (Silverwood et al., 2023), encouragement of physical movements during pregnancy (Gaston & Prapavessis, 2012), improvement of sleep quality (Paulino et al., 2022) and nonmedical interventions for gestational diabetes (Igwesi‐Chidobe et al., 2022). Research by Silverwood et al. (2023) illustrated groupings of interventions that have been observed in managing pregnancy well‐being, which include peer support components, psychological therapies and mind–body interventions. A recent review by Domínguez‐Solís et al. (2021) analysing anxiety reduction during pregnancy, postpartum and labour using nonpharmacological methods reported that expectant mothers were most likely to choose this method of coping and management of symptoms as they believe these methods have limited side effects for themselves and their baby. However, in terms of evidence quality, is it worth highlighting that significant effects of therapeutic methods on prenatal anxiety as reviewed by Domínguez‐Solís et al. (2021) were also met with more recent work by Paulino et al. (2022) who indicated that while interventions such as relaxation techniques and physical exercises had significant influences of women's sleep quality during pregnancy, the quality of existing studies were considered to be low overall. Therefore, continuous in‐depth measurement and analysis of long‐term effectiveness of these interventions on this demographic is vital.

Coping styles can be divided into two: positive and negative coping (Lau et al., 2015). Positive coping is the ability to effectively cope during stressful experiences by utilising problem‐focussed and active coping styles, which results in successful meaning‐making and emotion regulation. Conversely, negative coping style is more passive and emotion‐focussed, which results in limited abilities to emotionally communicate and rumination (Lau et al., 2015). The effects of these positive and negative coping approaches were also consistent with studies on positive psychology, as evidenced in a population of 915 pregnant women, in whom positive coping styles were found to have direct effects on perceived stress and depressive symptoms. Positive coping styles were further explained as comprising traits such as adapting quickly to difficult situations or attempting to reframe negative circumstances in a positive way (Lau et al., 2015). Yu and team (2020) reported consistent results when examining positive coping from pregnancy to 6 weeks postpartum with pregnant women engaging in more positive coping than negative, resulting in lower experiences of postpartum depression. However, coping strategies are not inherently ‘positive’ or ‘negative’; their adaptiveness depends largely on context, individual characteristics and stressor type, such as education, habits or individual's perception of stressors (Alves et al., 2023; Taubman–Ben‐Ari et al., 2021). Therefore, interventions that enhance flexible coping, allowing individuals to select strategies suited to their situation, are most likely to improve psychological outcomes during pregnancy.

PPI for the use and benefit of pregnant women is a small but growing scholarly pursuit. To highlight specific forms of positive coping, hope is one component within positive psychology that has been previously investigated with pregnant women (Delale et al., 2021; Noroozi et al., 2020; Samavi et al., 2018). Peterson and Seligman (2004) defined hope, similar to optimism as a trait that signifies a future‐oriented mindset from an emotional, cognitive and motivational perspective. In one study, hope and resilience were significantly correlated to increased attachment of mothers to their foetus during pregnancy (Noroozi et al., 2020), whereas hope was also found to be a significant predictor of quality of life (QoL) in Croatian pregnant women (Delale et al., 2021). Hope was also used as a positive psychology variable to curate a group‐based hope therapy to address mental health and labour pain (Samavi et al., 2018). They found that in a total population of 36 pregnant women divided equally into an intervention versus control study, hope therapy significantly reduced labour pain and increased positive emotions during pregnancy. Similarly, when it came to the role of optimism, a systematic review that synthesised two studies on obstetrical outcomes via the Life Orientation Trait‐Revised (LOT‐R) measure found that women with higher levels of optimism showed lower rates of preterm birth (Giangiordano et al., 2020).

In a narrative review of PPIs during the pregnancy period, Corno et al. (2019) found that gratitude was the most common PPI component that was included in interventions, which subsequently increased mental well‐being and reduced the risk of developing postpartum depression. With increasing medical and social costs for birth‐related mental health challenges, such as postpartum depression (Larsson, 2009), Drozd et al. (2015) highlighted the importance of amplifying outreach with pregnant women to provide accessible and evidence‐based support structures that can reduce risk of developing mental health issues in addition to equipping them with effective coping skills.

To the best of our knowledge, there is no systematic review on the application of positive psychology for pregnant women, besides a narrative review on PPIs during the perinatal period by Corno et al. (2019) and a systematic review performed to address one specific aspect of PPI, which is coping during pregnancy, by Guardino and Schetter (2014). The present review considered the detailed application of PPIs that have been specifically developed for pregnant women. The main research question for this review was ‘How do positive psychology interventions (PPIs) benefit the well‐being of pregnant women?’ Given the considerable heterogeneity across the studies, a narrative synthesis is valuable in identifying patterns across intervention components, theorising potential mechanisms and mapping specific PPI components to observed outcomes.

A preprint version of this manuscript is publicly available prior to peer review on the Open Science Framework repository (M.S Ganesan et al., 2024).

## METHODS

### Search protocol and registration

The protocol for this systematic review was registered following the International Prospective Registry of Systematic Reviews (PROSPERO), registration number (CRD42021257709; https://www.crd.york.ac.uk/prospero/display_record.php?RecordID=257709).

### Search strategy

The review was guided and conducted by the Preferred Reporting Items for Systematic Reviews and Meta‐Analyses (PRISMA) statement and checklist (PRISMA, 2023). Multiple search strings and suitable databases were identified (Author 1) and cross‐checked with a second reviewer (Author 2). As different search strings yielded different results, formal searches were conducted by changing search strings using the same keywords according to search formats and sensitivity of different databases

Electronic searches of literature studies published up to December 2023 were drawn from five databases: EBSCOhost, PsychINFO, Web of Science, PubMed and Scopus. Manual searches were also conducted on positive psychology journals, following the approach of a recent systematic review on positive psychology interventions (Carr et al., 2021). The manual searches were conducted in the *Journal of Positive Psychology*, *Journal of Happiness Studies*, *Journal of Positive Psychology and Well‐being*, and the *International Journal of Applied Positive Psychology*. Additionally, reference lists were checked in the full‐text articles accepted from finalised databases and positive psychology intervention systematic reviews.

We adapted and modified search terms from the narrative review conducted by Corno et al. (2019) for our catalogue of online databases, accordingly. Table 1 lists the main key words used.

**TABLE 1:** Primary key terms utilised for each database prefilters.

Online databases	Key terms
PsychInfo	all((pregnan* OR prenatal)) AND all((positive psychology)) AND all((interventions OR treatment))all((pregnan* OR prenatal)) AND all((positive psychology)) AND all((interventions OR treatment))
Web of Science	all = ((pregnan*ORprenatal))AND all = ((positive psychology)) AND all = ((interventions OR treatment))
EBSCOhost	Positive psychology AND (interventions or strategies or best practices or treatment or therapy or program or management) AND (pregnancy or pregnant or prenatal or antenatal or perinatal or maternal)
Scopus	ALL (‘positive psychology intervention’) AND ALL(‘pregnancy’) OR ALL(‘prenatal’)
PubMed	(((pregnancy) AND (positive psychology)) AND (intervention))

### Inclusion criteria

Papers could be observational intervention studies that may not include a specific comparator or control, as well as pilot and usability papers of positive psychology interventions within the population. Where appropriate to the design, studies with any type of comparator intervention or control group were included, including waitlist, treatments as usual and active control groups. Studies were limited to English language publications only. The research question and selection criteria were framed using the Population, Intervention, Comparison, Outcome and Study design (PICOS) formulation (Table 2). Additionally, selected studies that include information on spouses/fathers as part of their intervention were extracted.

**TABLE 2:** Selection criteria using PICOS.

PICOS formulation	Description
Participants	Pregnant women aged above 18 years, with or without any pregnancy‐induced physical or/and mental health conditions. This will provide a holistic understanding on the influence of positive psychology interventions on pregnant women's overall health. Key terms include pregnant, pregnancy, perinatal, prenatal, antenatal or/and maternal.
Interventions	Mainly included explicitly labelled positive psychology interventions (PPIs) aligned with positive psychology theories with the objective of improving well‐being during pregnancy, in any or both physical and psychological well‐being. Key terms included positive psychology, positive psychology interventions or treatment or strategies, for example.
Comparisons	Consist of control groups (if available according to study design), no intervention, or any other interventions besides positive psychology interventions.
Outcomes	Observations from included results of interventions on any changes in physical or/and psychological well‐being of pregnant women. Key terms observed were physical well‐being, physical health, health outcomes, maternal health, psychological well‐being, psychological health and mental health.
Study	All types of study labelled or referred to as a positive psychology intervention (PPI) aligned with positive psychology theories, offered for pregnant women. Studies could be quantitative, qualitative, mixed‐methods, pilot, observational and interviews, with no comparator/control or appropriate interventional designs including waitlist.

### Exclusion criteria

Owing to unique stressors experienced by different demographics of pregnant women, studies conducted within teenage/adolescent pregnancies, surrogate expectant women, pregnancies from in‐vitro fertilisation (IVF) or fertility‐related challenges and HIV positive pregnancy were excluded from our searches. Similarly, research on miscarriages and abortions was also omitted. Pregnancy and positive psychology interventions that were directly targeted to health behaviour changes, such as alcohol consumption or cigarette smoking, were not included. Lastly, studies that solely assessed the effects of mindfulness‐based interventions for this population were excluded (multiple systematic reviews have considered this, encompassing diverse applications and effects of mindfulness during pregnancy, specifically for perinatal well‐being) (Dhillon et al., 2017; Hall et al., 2016; Leng et al., 2023; Mao et al., 2023; Matvienko‐Sikar and Dockray, 2017), whereas mindfulness included as part of multi‐component positive psychology interventions was included. Multi‐component PPIs are interventions that incorporated two or more positive psychology components, such as gratitude, hope, strengths or mindfulness, combined. We acknowledge the possible introduction of selection and attribution bias when excluding studies that examined mindfulness as a single‐component intervention, which may consequently inflate observed effect sizes. Therefore, this systematic review focusses on interventions specifically designed to further understand the role of multiple positive psychological resources on pregnancy.

### Study selection

Results from all searched databases were exported to Zotero, a reference management software, and duplicates were removed. The first reviewer (Author 1) screened articles by title and abstract. Following this, two reviewers (Authors 1 and 2) screened full‐text articles against the inclusion and exclusion criteria. At the final selection stage, Authors 1 and 2 achieved consensus on the final list of selected studies by discussing any discrepancies towards the objectives of this review.

### Data management

Data extraction of specific study characteristics (Table 3) was performed by Author 1. We used a narrative synthesis approach to extract findings from each study and collate and summarise these findings thematically. Examples of categorisations included demographics and population, pregnancy information, physical health information, psychological health information, type of intervention, comparator (if available), main outcomes, additional outcomes, findings and specific highlights of each study. Details of extracted data were shared with Author 2 progressively. A discussion with second and third reviewers (Authors 2 and 3) followed to resolve any discrepancies and to triangulate the appraisal process. Meta‐analysis was not conducted because of study heterogeneity.

**TABLE 3:** Characteristics of included seven peer‐reviewed studies and one doctoral thesis.

Author(s) and study design	Country	Sample	Pregnancy stage	Age range	Positive Psychology Intervention (PPI)	Comparator	Main outcomes	Limitations
Jafari et al., 2022 (Quasi‐experimental)	Iran	*N* = 45 pregnant women with anxiety CBT *N* = 15 PPT *N* = 15 Control group *N* = 15	Second trimester	20–35 years old	Positive psychology training (PPT) for eight sessions, lasting 1 h each. Training starts with an introduction to PPT, overview of anxiety during pregnancy. Then, two main positive psychology constructs—‘strengths theory’ and ‘broaden‐and‐build theory’—are used to explain the intervention. Writing stories as part of a positive characteristics building exercise and later, gratitude activity using letter writing were two subsequent training sessions. Other training components focussed on life satisfaction, hope, optimism, habit building to increase positive feelings and concluded with a feedback session.	Cognitive behavioural therapy (CBT) and a control group.	There were significant results in both CBT and PPT versus the control group in analysing pregnancy anxiety, QoL, anxiety sensitivity and resilience. No significant differences between PPT and CBT on pregnancy anxiety.	Duration of study to analyse further effects of experiments. Pregnant women's emotions when answering self‐reporting questionnaires; to include follow‐up interviews.
Corno et al., 2017 (Case series)	Spain	*N* = 6	No specific gestational weeks required, women who intend to keep their pregnancy	18–41 years old	‘Positive pregnancy’ – web‐based, self‐help tool. Intervention lasts for 5 weeks, and it is performed online. Positive psychology components highlighted as part of this intervention specifically for pregnant women consist of mindfulness, savouring, connectedness for social support improvement and two sessions for both optimism and life satisfaction.		Expectant mothers reported improvement in psychological health from pre‐ to post‐test, reduction of depressive symptoms and pregnancy‐related anxiety while satisfaction with life was reported to increase from baseline to post‐intervention.	Varied gestational weeks/trimesters of participants resulting in possible different current experiences of pregnancy.
Haga et al., 2020 (Randomised controlled trial)	Eastern Norway	*N* = 1342 Intervention (*N* = 678) Control (*N* = 664)	21–37 weeks	18 years and above	The intervention – Mamma Mia has a total of 44 sessions that runs for a duration of 11.5 months. Intervention was conducted to improve subjective well‐being of pregnant women. Components included mindfulness, gratitude and acts of kindness. Intervention was also conducted thrice at postpartum stage.	Control group (general pregnancy care)	Negative emotions were reduced in the intervention group comparatively; however, no significant difference found for satisfaction with life and positive emotion levels in pregnant women.	Higher participant drop‐out in the intervention group than control. Higher scores of life satisfaction and positive affect at baseline reducing possible effects of intervention.
Haga et al., 2018 (Randomised controlled trial)	Eastern Norway	1342 Intervention (*N* = 678) Control (*N* = 664)	21–37 weeks	18 years and above	The intervention – Mamma Mia has a total of 44 sessions that runs for a duration of 11.5 months from 21 weeks pregnancy to 6 months postpartum. Intervention was conducted to improve perinatal depressive symptoms in pregnant women.	Control group (general pregnancy care)	Results were significant for Mamma Mia participants in improvement of depressive symptoms. The prevalence of higher depressive scores were lesser over follow‐up (time) sessions.	Participants with higher depressive scores had higher dropout rates.
Samavi et al., 2018 (Pre‐post‐test)	Iran	*N* = 36 Intervention (*N* = 18) Control (*N* = 18)	25 to 30 weeks	Unreported	Hope therapy was performed for 8 weeks with the aim of improving labour pain and psychological well‐being of pregnant women. Intervention consisted of an introduction to hope, goal setting, management and familiarity with labour and pain, and lastly, focus on mental well‐being.	Control group	Hope therapy was significantly effective to improve labour pain and mental health of pregnant women.	The study did not use specified questionnaires for various mental health variables.
Barber & Masters‐Awatere, 2022 (Mixed methods)	New Zealand	*N* = 88	24 to 36 gestation weeks	19–43 years old	The intervention – ‘positively pregnant’ was ran as a pilot study to learn the applicability of this mobile application to be utilised as a stress management tool (depression, anxiety and stress). Anchored in positive psychology strength‐based traits, the pilot version of the application consisted of four components with its own set of highlight activities; know yourself (e.g. stressors and emotions), do something (guided imagery and games), conversations (e.g. finances and caring for baby) and find out (e.g. stress and relationships).	N/A	Pilot participants reported significant improvement in managing stress with participants finding 23 out of the 26 specific activities from the intervention helpful.	Lack of qualitative evidence to provide further user experience of the mobile application. Pilot was examined at one time stamp with suggestion to test it across pregnancy duration.
Abbasi et al., 2022 (Randomised controlled trial)	Iran	*N* = 60 Control *N* = 30 Intervention *N* = 30	6–10 weeks pregnant	18–35 years old	Intervention participants were exposed to six supportive counselling sessions lasting 45 min each across three times per week with the aim to improve vomiting and nausea symptoms during pregnancy. The six sessions were namely: introduction to the 24‐character strengths, paths to happiness, positive emotions, gratitude, interpersonal relationships, hope, optimism and lastly a reflective session.	Control group (general care)	Coping scores significantly increased after 4 weeks of the intervention in the experimental group compared to control.	Owing to COVID‐19, study was unable to recruit pregnant women with severe nausea and vomiting symptoms in addition to pursuing a longer follow‐up study.
Matvienko‐Sikar & Dockray, 2017 (Randomised controlled trial)	Ireland	*N* = 36 Experiment *N* = 24 Control *N* = 12	10–22 gestational weeks	27–40 years old	This positive psychology intervention called ‘bundle of joy’ included two components: online mindfulness and gratitude exercises. Experiment ran for 3 weeks, with four sessions a week. Measurements of prenatal stress, cortisol and life satisfaction levels were taken three times (baseline, 1.5 and 3 weeks)	Control (general care)	Prenatal stress was significantly reduced in trial group comparatively with specific significant improvements only shown in well‐being domains of waking and evening cortisol levels.	Small sample size. No qualitative data collected to further pursue participants' experiences with the intervention for future improvement.
O'Leary, pp 304, 2016 (PhD Thesis) (Randomised controlled trial)	Ireland Norway	*N* = 62 Intervention 1, exposed to mindfulness exercise first: (*N* = 17) Intervention 2, exposed to gratitude diary task first: (*N* = 15) Control: (*N* = 14)	10 to 22 weeks gestational weeks	27 to 40 years old	The intervention – ‘bundle of joy’, an online assessment were interested to measure subjective well‐being (life satisfaction, subjective happiness, perceived social support, prenatal stress), physical well‐being (sleep quality, birth outcomes), mental health (depression) and cortisol levels using mindfulness meditation and gratitude diary. Cortisol saliva samples were collected at the 1.5‐ and 3‐weeks mark with the intervention utilised for 3 weeks, four times per week.	Control	Only sleep quality was significantly improved from this intervention study.	Between the gratitude diary and mindfulness meditation, only the prior was able to be tracked by researchers for completion rates.

### Methodological assessment

Methodological quality was evaluated using two main quality assessment tools. Case series, quasi‐experimental and randomised control trials were assessed using the Joanna Briggs Institute (JBI) appraisal tool (Joanna Briggs Institute, 2017). In the JBI case series checklist, 10 questions with ‘yes’, ‘no’, ‘unclear’ and ‘not applicable’ as options were provided. Examples of questions include the use of appropriate statistical analysis, outcomes of follow up results and clear write‐up on inclusion criteria. In a similar layout, the quasi‐experimental version of the JBI checklist included nine questions with the same four options. Here, questions included the presence of a control group, definite cause and effect for the study and nature of the follow‐up process. The final JBI appraisal tool was one used for randomised controlled trials (RCTs), which contained three main sections: internal validity, participant retention‐related bias and validity of conclusion derived from statistical analysis. Mixed‐methods studies were assessed by the McGill Mixed Methods Appraisal Tool (MMAT) (Hong et al., 2018). The tool consists of two parts: checklist and criteria explaining. In the checklist category, authors select one type of study design and subsequently answer five questions with given responses: ‘yes’, ‘no’ and ‘cannot tell’ followed by a comment section. Similarly in the criteria part, each of the questions answered earlier is supplemented with a short methodological explanation. The methodological quality assessment process was independently conducted by the two main reviewers (Authors 1 and 2) with discussion to resolve any discrepancies.

## RESULTS

### Selected studies

**FIGURE 1:**
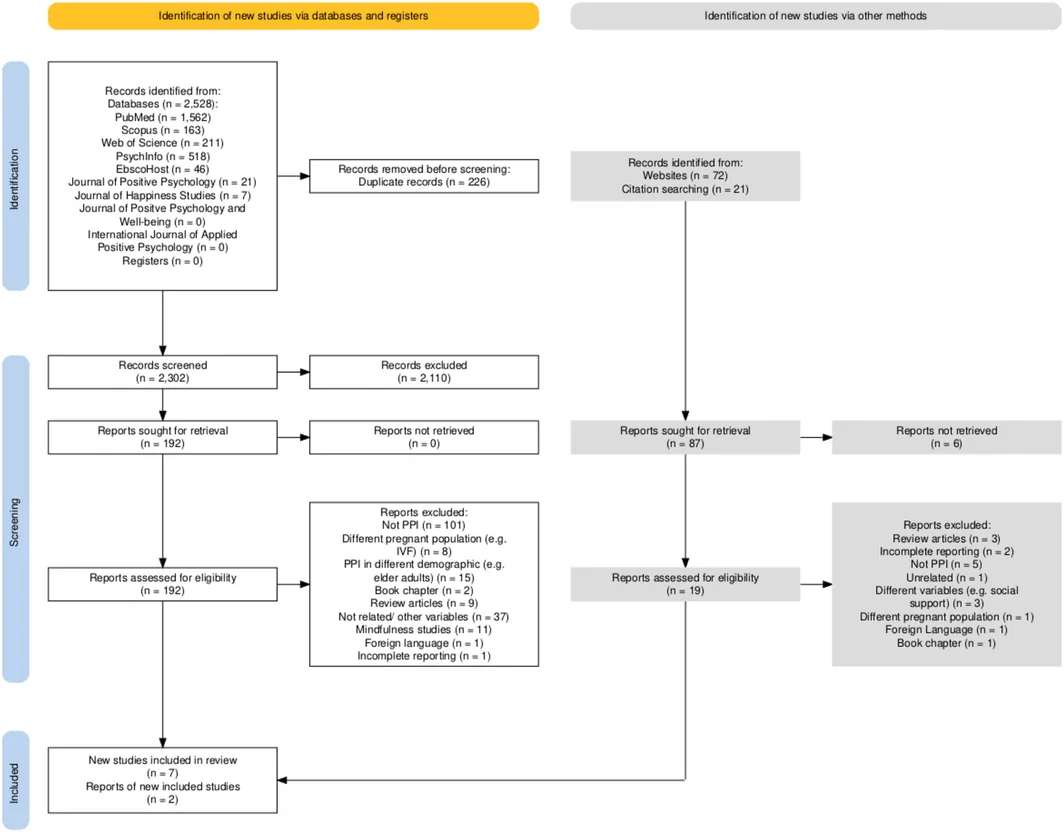
PRISMA flow chart for recording of searches.

The PRISMA chart produced from Haddaway et al. (2022) (Figure 1) illustrates the review process. Initial search from databases retrieved 2528 records. After 226 duplicates were removed, the remaining 2302 records proceeded to the title and abstract screening stage. Following this, 192 records were retained for full‐text screening. After the exclusion of 185 records, seven were included in this review.

Records were also searched via other methods; 72 records were found on literature mapping websites and 21 records through citation searching. Six duplicates were removed, allowing 87 records to be screened as mentioned earlier. Ultimately, two records were retained from this route.

In total, nine records were included, comprising eight peer‐reviewed records and one doctoral thesis. As two peer‐reviewed records were based on the same ‘Mamma Mia’ intervention, these were therefore counted as one study throughout this review. The resulting eight studies included in this review were narratively synthesised in Table 3.

### Main findings

#### Effect sizes

Based on effect size calculations, Jafari et al.'s (2022) intervention explained a large effect size across psychological outcomes (partial *η* ^2^ = 0.571, *p* < .001). Samavi et al. (2018) reported a large effect size for labour pain (Cohen's *d* ≈ 1.77) based on post‐test differences (*t* = 5.17, *p* < .001) and a large effect for mental health (partial *η* ^2^ = 0.46; Cohen's *d* ≈ 1.85). Barber and Masters‐Awatere (2022) found a significant reduction in subjective stress (*η* ^2^ = 0.088, *p* = .023), indicating that approximately 8.8% of variance in stress scores was attributable to app use, Cohen's *d* of ≈0.60, suggesting a medium intervention effect on stress. Abbasi et al. (2022) found supportive counselling to show significant interaction with partial *η* ^2^ = 0.43, corresponding to a large effect (Cohen's *d* ≈ 1.7). Corno et al. (2017), Haga et al. (2019, 2020) and Matvienko‐Sikar and Dockray (2017) did not report sufficient summary statistics to allow the calculation of standardised effect size for prenatal well‐being outcomes.

#### Study characteristics

Eight studies with varied designs were included in this review. The selection comprised of one quasi‐experimental, one case series, one pre–post‐test, three RCTs, one pilot RCT and one mixed methods. The smallest sample was six for a case series (Corno et al., 2017) and the largest 1342 participants for the RCT (Haga et al., 2020, 2018). The rest of the studies reported between 36 and 103 participants (Abbasi et al., 2022; Barber & Masters‐Awatere, 2022; Jafari et al., 2022; Matvienko‐Sikar and Dockray, 2017; Samavi et al., 2018).

The Mamma Mia intervention reported ethnicity of pregnancy sample, which were namely Eastern Norwegian (Haga et al., 2018, 2020) and a mixed‐race sample based in New Zealand representing New Zealand European, New Zealand Māori, Pacific, Asian, other European and other races (Barber & Masters‐Awatere, 2022). Finally, majority of participants in O'Leary's (2015) and Samavi et al.'s (2018) studies were reported to be from Irish and Iranian demographics, respectively.

The most investigated outcomes for pregnant women were depression, subjective well‐being, stress, anxiety and emotions. Mamma Mia investigated well‐being outcomes inclusive of the postpartum period (Haga et al., 2018, 2020). Of the eight studies, only one had specific inclusion criteria for health characteristics: participants with nausea and vomiting symptoms (Abbasi et al., 2022), with the others being tested in a general pregnancy population. The most utilised positive psychology domains for development or exposure of intervention to improve well‐being during pregnancy were subjective well‐being (life satisfaction, optimism, hope), social support, gratitude, emotions and sense of meaning.

### Contents of PPIs

Seven interventions were included within the selected studies that are aligned with the origins of positive psychology. Firstly, Mamma Mia, a web‐based intervention was initially conducted in a pilot study focussed only on the intervention content outcomes (Haga et al., 2013) and two reports of the RCT (Haga et al., 2018, 2020); both RCTs were counted in this review as one study. Other therapies and interventions used included group hope therapy (Samavi et al., 2018), a combination of cognitive behavioural and positive psychology training (Jafari et al., 2022), ‘positive pregnancy’, also a web‐based intervention (Corno et al., 2017), ‘positively parent’, a mobile application for well‐being in pregnancy (Barber & Masters‐Awatere, 2022), and supportive counselling using positive psychology approaches (Abbasi et al., 2022).

#### Group hope therapy (Samavi et al., 2018)

Samavi et al.'s (2018) study with pregnant Iranian women focussed on the elevation of hope levels via group therapy and the consequent effects on labour pain and psychological health. The framework of their group hope therapy was based on Snyder (1999), which encompasses four parts to its 90‐min therapy session for participants in the experimental group. There were 13 guiding points that were discussed in these sessions. Examples include introducing hope and its components to the participants, discussing the challenges and pain experienced by pregnant women, and changing negative inner dialogues. As the hope therapy was conducted for 8 weeks, a brief overview of the 90‐min session comprised of a reflection of tasks performed in the previous week for the first 20 min. The participants learn to enhance their hope skills for the categories of goals, paths and agency—the main points of hope conceptualised by Snyder (1999) in the second 20 min. This is followed by a longer session of 40 min to learn application methods of the hope skills in real‐life contexts by sharing respective experiences and using them to manage labour pain. In the final 10 min, the participants are made aware of activities in the following session. Experimental groups were also provided with take home guides and activities to further improve their hope practice. Well‐being domains that were assessed in this research were physical health, labour pain and mental health assessment measuring physical symptoms, depression, anxiety, and insomnia plus social dysfunction. Hope therapy was found to be significant both in the analysis of labour pain and mental health by reducing pain levels and improving mental well‐being. This was the only study identified that employed hope as its main positive psychology intervention component; therefore, no definitive conclusions can be drawn.

#### Positive psychology training (Jafari et al., 2022)

In another Iranian study, Jafari and colleagues (2022) assessed the effectiveness of cognitive behavioural training (CBT), PPT and a control group on psychological well‐being for second trimester pregnant women. Their PPT module was adapted based on a therapist's' guide for positive psychology interventions developed by Magyar‐Moe (2009). The PPT was conducted for 1 h per session, weekly, a total of eight times. The first session consists of an introduction to and an understanding of anxiety and its roots. Positive psychology was introduced to participants in the second session, which highlighted two main concepts (strengths theory and broaden‐and‐build theory of positive emotions). A ‘positive introduction worksheet’ is used in the third session for participants active engagement towards writing a positive self‐introduction and was guided by trainers to identify positive personal characteristics. Gratitude exercises were administered at the fourth session, followed by another exercise to learn and increase pregnant women's life satisfaction levels. Hope and optimism were elements introduced at the sixth session using the ‘closed door–open door’ perspective. The seventh session had a mixture of activities such as ‘savouring’ activities and the importance of understanding that cultivating a continuous ‘habit’ towards favourable tasks may reduce its consequent positive impact. At the final PPT session, the participants discussed barriers, facilitators and perspectives of the whole training; additionally, they were given guidance by the trainer.

In the comparison group, CBT was administered, utilising a previously examined intervention based on Hossein Khanzadeh et al., (2017) work. The duration and frequency of sessions remained constant as the PPT. The control group was not provided any intervention exposure.

Psychological well‐being variables assessed were anxiety sensitivity, prenatal distress, quality of life and resilience. Both the PPT and CBT groups reported significant benefits across all measures versus the control group, although there were no significant differences between PPT and CBT.

#### Mamma Mia: RCT for perinatal depression (Haga et al., 2018) and subjective well‐being (Haga et al., 2020)

Mamma Mia was a web‐based intervention specifically for pregnancy with three main objectives: to prevent postpartum depression, to provide self‐help treatment for mild‐to‐moderate depression during pregnancy and for new mothers and to enhance overall subjective well‐being (Haga et al., 2020). Conceptualised by a combination of three schools of thought, Mamma Mia employs components from positive psychology, couples therapy and metacognitive therapy. This was visible in the complete list of contents within the web‐based intervention, which constituted eight items. Firstly, a depressive assessment, followed by metacognitive therapy and then a positive psychology session. The fourth session was allocated to couples therapy, whereas the fifth and sixth sessions were breastfeeding and psychoeducation activities. Mamma Mia was conducted for a total of 44 sessions across three phases (pregnancy, active, follow‐up phases) with 11 sessions specifically conducted in the first phase, pregnancy stage, starting at 18–24 gestational weeks to 36–40 gestational weeks depending on participants estimated due date. All of these sessions are administered via email and a website, with no face‐to‐face sessions.

The positive psychology component in this intervention provided opportunity for women to engage in multiple activities anchored by existing frameworks founded in Peterson and Seligman's (2004) work. Description of activities that pregnant women engaged with included a gratitude exercise to reflect on individual blessings, socialisation to increase appreciation and access towards social support by encouraging an active task of inviting a friend out, inspiring acts of kindness, envisioning one's future life goals and matching them with dreams that have come true to increase optimistic thought processes, and lastly, a task on pleasant activities conceptualised by CBT evidence.

The first Mamma Mia report (Haga et al., 2018) informed effectiveness on depressive symptoms and associated moderating variables such as parity and experience of depression in the past. The participants were administered the intervention from pregnancy weeks 21 to 25 and at week 37, while the postnatal period encompassed 6 weeks, 3 and 6 months post‐delivery. Depression levels significantly reduced for the intervention group, specifically at pregnancy week 37 and 6 weeks post‐delivery, compared to the control group. A reduction of 21.5%–26.6% of minor depression incidence was observed in the intervention group from gestational week 37 onwards. The researchers found that the participants with a higher depression score at baseline benefited more.

In the final publicly available evidence (Haga et al., 2020) of the Mamma Mia intervention, the data on subjective well‐being improvement during the perinatal period from the same group of participants in the study above were analysed. Similarly here, they investigated three outcomes: efficiency of Mamma Mia as a self‐help internet intervention for subjective well‐being experiences in pregnant women, effectiveness over time and associated moderators. Subjective well‐being components were satisfaction with life and emotions. For emotions, Mamma Mia did not yield any significant changes for positive affect; however, there were significant reductions in negative affect especially at pregnancy week 37, 1.5 months and 6 months post‐delivery. There were no significant effects on life satisfaction.

#### Positively pregnant: mobile application (pilot) (Barber & Masters‐Awatere, 2022)

‘Positively pregnant’ is a mobile e‐health application (Barber & Masters‐Awatere, 2022) anchored in the philosophy of positive psychology, namely, the broaden‐and‐build theory that focusses on the influences of positive emotions on one's well‐being (Fredrickson, 2001) and the dual‐system model (Wong, 2011) combining value and meaning‐making processes with cultural intricacies to attain a good life. There are four main parts to this mobile application that address different information: (1) ‘know yourself’ collects information on stressors, social support and health behaviours; (2) ‘do something’ encompasses active tasks like guided imagery, goal‐setting and journaling; (3) ‘conversations’ addresses aspirations and preparation towards the birth and arrival of their newborn, traditional customs and finances; and (4) ‘find out’ supports psychological preparation for the change and aspects of parenthood transition such as change in relationships and taking care of their well‐being. The participants in this study were recruited if they were less than 6 months pregnant, with both first‐time and experienced expectant mothers accepted. Well‐being measures that were examined were depression, anxiety and stress at baseline, 24 weeks and follow‐up at 36 weeks. Significant results were only produced for the stress outcome across time with the authors attributing lower levels of anxiety and depression reported at baseline to observe a significant change across time from baseline to 36 weeks. As this was a pilot study, there was no control group for formal comparisons.

#### Gratitude and mindfulness based interventions (Matvienko‐Sikar & Dockray, 2017)

Studies that conducted gratitude and mindfulness‐based interventions were previously reported in the narrative review by Corno et al. (2019). Matvienko‐Sikar and Dockray (2017) investigated a pilot on stress during pregnancy and subjective well‐being, while O'Leary's (2015) doctoral thesis included a RCT on the effects of the positive psychology intervention for pregnant women's psychological, physical and subjective well‐being. Both studies utilised similar methodologies for their respective participant groups and were administered a gratitude diary in which participants reflected and listed five elements for which they were most grateful for, as well as mindfulness exercise via an audio file that focussed on their breathing. The participants (*n* = 36) in O'Leary's (2015) and (*n* = 46) in Matvienko‐Sikar and Dockray (2017) were between gestational weeks 10 and 22 and could not have a prior mental or physical health diagnosis. Cortisol levels were used to quantify prenatal stress three times in both studies: at baseline, 10.5 and 21 days later. Results of the studies found that the dual‐component intervention significantly reduced prenatal stress compared to the control group, with both waking and evening cortisol levels showing improvement. Other well‐being components tested, such as depression, life satisfaction, gratitude and mindfulness, did not show any intervention‐associated changes. Most participants were well educated and actively completing health‐conscious behaviours with higher levels of baseline stress levels; hence, researchers noted the missed opportunity to assess the intervention among those who may have found it more beneficial such as those from lower socio‐economic statuses (SES), lower education or with poor health.

#### Positive pregnancy: virtual intervention (case series) (Corno et al., 2017)

Six pregnant women were included in this case series research by Corno et al. (2017) to understand the effects of a 5‐week web‐based positive psychology exercise on perinatal well‐being. Well‐being aspects assessed were subjective well‐being, depression, worries towards overall health of mother and child, in addition to childbirth and newborn concerns, and finally, perceived social support levels. Modules of the positive psychology programme that ran across 5 weeks included mindfulness and self‐acceptance, savouring, connectedness, and 2 weeks of optimism and life satisfaction modules. Each participants' pre‐ and post‐scores were analysed; all participants showed improvement for depression and life satisfaction with exception of one participant for prenatal anxiety. The most preferred psychology exercise was the ‘baby steps exercise’, included in part two of the optimism and life satisfaction module, where participants are requested to list goals that they would like to achieve and their initial ‘baby steps’ towards accomplishing them.

#### Supportive counselling using positive psychology (Abbasi et al., 2022)

Abbasi et al. (2022) utilised a supportive counselling intervention specifically for coping with nausea and vomiting symptoms in pregnant women based on a protocol by Seligman et al. (2006). Six counselling sessions were conducted for participants ranging between 45 and 60 min per session. The first session constituted an introduction to positive psychology based on the 24‐character strengths, followed by understanding physiological changes during pregnancy; in this context, vomiting and nausea were the focus. Next, the participants learnt happiness for stress reduction and adaptability to stressors and taught the role and applicability of positive and negative emotions in sessions two and three, respectively. The fourth session included processing forgiveness and practising gratitude; improving social relationships, optimism and hope were highlights of session five, and the sixth session was a reflective opportunity to recollect the previous exercises. Take home exercises were provided to further develop these positive behaviours, with a follow‐up analysed at the 4‐week mark. This study was conducted during the COVID‐19 period using a one‐on‐one format. Improvement of physical health in pregnant women was the main interest, specifically coping strategies for nausea and vomiting. There were two measures in this study: coping techniques during pregnancy, and nausea and vomiting severity. As a result of this intervention, the analysis produced significant change across intervention versus control groups and time for both outcome measures.

### Subgroup summary of main positive psychology domains

As described earlier, the included studies encompassed a range of PPI that were curated based on several theoretical positive psychology domains such as hope, gratitude, strengths‐based, meaning‐centred or mixed approaches (Table 4). A narrative synthesis suggests that gratitude and strengths‐based domains were applied more in both single and multi‐approach interventions. These domains evidenced significant improvements in outcomes of depression, prenatal distress, negative emotions and nausea and vomiting symptoms. Considering the small number of studies with varied outcomes and positive psychology domains used here, future research could consider standardised intervention components suitable for PPIs during pregnancy to encourage comparisons across studies.

**TABLE 4:** Subgroup of positive psychology domains utilised within each intervention.

Positive psychology domain	No. of studies	Primary outcomes	Observed effects
Hope	1 (Samavi et al., 2018)	Labour pain and psychological health	Improved labour pain and mental health
Strengths‐based and gratitude	2 (Jafari et al., 2022; Abbasi et al., 2022)	Psychological health (anxiety, prenatal distress) Nausea and vomiting symptoms	Improved mental health Improved coping techniques towards physical health symptoms
Gratitude exercise	2 (Haga et al., 2018; Haga et al., 2020)	Depression Subjective well‐being (Life satisfaction and emotions)	Improved depression levels No significant effects on life satisfaction observed while negative emotions were significantly reduced.
Positive emotions (broaden‐and build theory)	1 (Barber & Masters‐Awatere, 2022)	Depression, anxiety, stress	Only stress levels improved
Gratitude and mindfulness	1 (Matvienko‐Sikar & Dockray, 2017)	Prenatal stress, depression, anxiety, life satisfaction	Prenatal stress improved
Self‐acceptance, optimism and mindfulness	1 (Corno et al., 2017)	Life satisfaction, depression, worries of overall health, childbirth concerns and perceived social support	Depression and life satisfaction levels improved

### Summary of instruments used to measure well‐being in PPIs studies

Studies utilised various validated self‐administered questionnaires to measure outcomes such as physical well‐being, mental health, subjective well‐being and coping abilities. The instruments are listed in Table 5 according to respective selected studies.

**TABLE 5:** List of instruments utilised in selected studies.

Outcomes/variables	Validated measures	Study
Mental well‐being	General Health Questionnaire (GHQ) (Goldberg & Williams, 1988)	Samavi et al., 2018
Anxiety sensitivity	Anxiety Sensitivity Index (ASI) (Reiss et al., 1986)	Jafari et al., 2022
Distress	Depression, Anxiety and Stress Scale (DASS‐21; Lovibond & Lovibond, 1995; Henry & Crawford, 2005)	Barber & Masters‐Awatere, 2022
Quality of life	World Health Organisation Quality of Life (WHOQOL) (Webster et al., 2010)	Jafari et al., 2022
Resilience	Connor–Davidson Resilience Scale (CD‐RISC) (Kathryn et al., 2003)	Jafari et al., 2022
Subjective well‐being	Satisfaction with Life Scale (Diener et al., 1985)	Haga et al., 2020; Matvienko‐Sikar & Dockray, 2017
Emotions	Positive and Negative Affect Schedule (Watson et al., 1988)	Haga et al., 2020
Mindfulness	Mindfulness Attention Awareness Scale (MAAS) (Brown & Ryan, 2003)	O'Leary, 2015; Matvienko‐Sikar & Dockray, 2017
Subjective happiness	Subjective Happiness Scale (SHS; Lyubomirsky & Lepper, 1999)	O'Leary, 2015
Mental functioning and subjective well‐being	The Warwick–Edinburgh Mental Well‐Being Scale (WEMWBS25) (Tennant et al., 2007)	Corno et al., 2017
Sleep quality	Jenkins Sleep Questionnaire (JSQ; Jenkins et al., 1988)	O'Leary, 2015
Perceived social support	The Multidimensional Scale of Perceived Social Support (MSPSS30) (Zimet et al., 1988)	Corno et al., 2017
Labour pain	Visual Analog Scale (VAS) (Bijur et al., 2001)	Samavi et al., 2018
Depression during pregnancy and postnatal period	Edinburgh Postnatal Depression Scale (Cox et al., 1987) The Patient Health Questionnaire‐9 (PHQ‐926) (Kroenke & Spitzer, 2002)	Cox et al., 1987; Matvienko‐Sikar & Dockray, 2017 Corno et al., 2017
Pregnancy related worries, stress	Prenatal Distress Questionnaire (PDQ) (Alderdice & Lynn, 2011) The Pregnancy Related Anxiety Scale (PRAS28) (Rini et al., 1999)	Jafari et al., 2022; Matvienko‐Sikar & Dockray, 2017 Corno et al., 2017
Coping methods during pregnancy	Revised Prenatal Coping Inventory (Hamilton & Lobel, 2008)	Abbasi et al., 2022
Frequency of nausea, vomiting and gagging experience in the last 12 h	Index of Nausea and Vomiting (Zhou et al., 2001)	Abbasi et al., 2022
Gratitude during pregnancy	Gratitude during Pregnancy (GDP); *developed and validated for the study* scale published in O' Leary et al. (2016)	O'Leary, 2015; Matvienko‐Sikar & Dockray, 2017

### Study quality

From the eight studies reviewed, there were 1675 pregnant women assessed to understand the usefulness of PPIs and their subsequent effects on well‐being. Two quasi‐experimental studies and five quantitative RCTs all had a control intervention group, which increased their internal validity. Information on participant dropout was reported in three studies that experienced loss to follow‐up (Haga et al., 2018, 2020; Matvienko‐Sikar and Dockray, 2017; Samavi et al., 2018), whereas Abbasi et al. (2022) stated no dropouts. Dropout rates for Haga et al.'s (2018 and 2020) studies were moderate at approximately 30.8%. Much lower dropout rates were reported by Matvienko‐Sikar and Dockray (2017) and Samavi et al. (2018), with 21.7% and 5% dropout, respectively. The participant characteristics associated with dropout included undergoing preterm labour during the course of intervention (Samavi et al., 2018), lower education and existing childcare commitments (Haga et al., 2020). Six studies had group allocations in their study (intervention versus control), whereas Barber and Masters‐Awatere (2022) and Corno et al. (2017) did not have a control condition. As listed in Table 5, all assessments in the selected studies employed validated measures; the Edinburgh Postnatal Depression Scale (EPDS; Cox et al., 1987) was the most administered survey, being used in half of the included studies (Barber & Masters‐Awatere, 2022; Haga et al., 2018; Matvienko‐Sikar and Dockray, 2017 and O'Leary, 2015). O'Leary (2015) developed and validated a positive psychology survey for specific use with pregnant women. The published survey, Gratitude during Pregnancy Scale (O' Leary et al., 2016), was later also used in one of the selected studies by Matvienko‐Sikar and Dockray (2017). With the heterogeneous research designs, a meta‐analysis to identify an overall effect size was not feasible.

## DISCUSSION

To our knowledge, this is the first review to synthesise the effects of positive psychology interventions during pregnancy comprising outcomes from both physical and psychological health. The following discussion provides an overview of the applied positive psychology theories and well‐being outcomes investigated in the selected studies. As a result of this systematic review, it was identified that PPI for maternal well‐being were aimed at improving (1) physical health, including labour pain, nausea and vomiting; (2) psychological health, including stress, emotions, anxiety and depression; and (3) subjective health, including life satisfaction and perceived social support.

Several interventions identified in this systematic review were anchored by established and pioneering positive psychology theories, such as the character strengths‐based theory (Peterson and Seligman, 2004), broaden‐and‐build theory (Fredrickson, 1998, 2001), hope theory (Snyder, 1999), positive psychotherapy (Seligman et al. 2006) and PPT (Magyar‐Moe, 2009). Barber and Masters‐Awatere (2022) used a newer positive psychology model, the dual‐system model of the good life (Wong, 2011), to be more inclusive towards culture in optimising the combination of positives and negatives. In expanding the application of existing theories, other studies (Corno et al., 2017; Jafari et al., 2022) used a combination of techniques and therapies (meaning‐making, letter writing, social connectedness) in curating their positive psychology interventions, proposed to increase the potential for positive well‐being in pregnant women. Likewise, the development of interventions by integrating positive psychology components with classical therapies was trialled in group hope therapy for labour pain and mental health by Samavi et al. (2018) and supportive counselling sessions for improvement of nausea and vomiting symptoms by Abbasi et al. (2022). We observed significant benefits on well‐being during the perinatal period across studies, highlighting the ability to improve well‐being or/and coping skills with specific theory‐informed interventions. That said, given the limited number of studies, there is a clear need for further research to better understand the applicability and effects of positive psychology interventions for pregnant women.

Although included studies employed multi‐component PPIs, the specific contributions of each component to outcomes have been synthesised. A component‐outcome mapping is listed in Table 6. A qualitative comparison of the reported effect sizes across the included studies reveals a clear pattern. PPIs produced large effects on self‐reported psychological outcomes, including mental health (Samavi et al., 2018) and coping with pregnancy (Abbasi et al., 2022). Similarly, interventions significantly reduced labour pain (Samavi et al., 2018), although a more physiological outcome may be influenced additionally by psychological perceptions. In contrast, depressive symptoms measured via EPDS (Haga et al., 2019) and subjective stress (Barber & Masters‐Awatere, 2022) demonstrated smaller or moderate effect sizes. Notably, some studies, such as Matvienko‐Sikar and Dockray (2017), reported significant improvements in prenatal stress but did not provide sufficient statistical details to calculate standardised effect sizes. Additionally, interventions that included social interaction may have leveraged social–emotional mechanisms, providing perceived support that contributes independently to coping and well‐being.

**TABLE 6:** Component‐outcome mapping of selected studies.

PPI component	Pathway	Outcomes	Studies
Gratitude exercises	Increased positive emotions	Subjective well‐being, emotions	Samavi et al. (2018); Abbasi et al. (2022)
Strengths exercises	Increased self‐efficacy and resilience	Coping, psychological well‐being, perceived mastery	Abbasi et al. (2022)
Optimism/hope‐building exercises	Increased positive expectancies	Perceived stress, life satisfaction	Samavi et al. (2018)
Mindfulness	Reduced rumination and stress levels, increased emotional regulation	Stress, depressive symptoms	Barber & Masters‐Awatere (2022); Haga et al. (2019)
Psychoeducation	Increased cognitive control	Stress reduction, coping	Matvienko‐Sikar and Dockray (2017)
Social support	Reduced isolation, increased perceive social support	Stress reduction, coping	Barber & Masters‐Awatere (2022); Matvienko‐Sikar & Dockray (2017)

These findings suggest testable hypotheses for future research. For example, increases in resilience or self‐efficacy levels may mediate improvements in psychological well‐being, whereas social support may mediate or moderate reductions in prenatal stress (social–emotional). Future trials could employ mediation, moderation or factorial designs to evaluate which specific PPI components contribute towards certain outcomes.

Specific positive psychology interventions yielded similar outcomes across different types of interventions, such as the PPT intervention in Jafari et al. (2022) and a web‐based self‐help intervention in Corno et al. (2017) with significant effects on reduction in pregnancy anxiety. Both these studies included hope and optimism as part of their intervention components; therefore, more studies should be conducted to understand the differences between the content of the interventions themselves and the effects among pregnant women when the intervention is present or absent. Conversely, while Corno et al. (2017) reported evidence of significant intervention effects for life satisfaction, Haga et al. (2020) and O'Leary (2015) did not. Examining the differences across the existing studies is important when developing future PPI with pregnant and postpartum women, for example, in considering factors such as the length or method of intervention. In this review, the shortest and longest duration were 3 weeks and 11 months accordingly, with most interventions administered between 3 and 8 weeks. As recommended by a previous review that focussed on the psychological well‐being during pregnancy (Corno et al., 2019), all studies identified were conducted for pregnancy well‐being, with only one study—the Mamma Mia intervention—extending into the postpartum phase. Given the limited pool of postpartum participants who have administered PPI, it is not currently possible to understand the difference in the impact of these interventions during pregnancy versus postpartum. The Mamma Mia intervention feasibility article (Haga et al., 2013) reported the recruitment of spouses/partners. Involvement of fathers could be further experimented to gain more insights into the benefits gained by fathers when included in the transition period.

We also note that many of the positive psychology interventions were conducted virtually by self‐help, web‐based platforms (Corno et al., 2017; Haga et al., 2018, 2020; O'Leary, 2015) or mobile applications (Barber & Masters‐Awatere, 2022). These digital interventions supported significant improvements in well‐being outcomes, including depression (Haga et al., 2018) and stress management (Barber & Masters‐Awatere, 2022), coupled with increasing accessibility and flexibility for some pregnant women. Virtually conducted positive psychology interventions, while still new and requiring more evidence, could be deemed a suitable alternative tool for women in advanced stages of pregnancy to access from the comfort of their home and comparatively a more efficient use of time when it comes to arranging multiple health appointments during the pregnancy and postpartum phase. This is supported by Jafari et al. (2022), who recruited only second trimester pregnancies in their training‐based study, as pregnant women in their first and third trimesters may experience challenges relating to physical health (nausea) or physical movement. Virtual tools would have been particularly beneficial during the COVID‐19 pandemic when pregnant women faced higher health risks from increased outdoor exposure. As a comparison, two studies (Boucher et al., 2021; Brouzos et al., 2021) that were excluded from the review as they were conducted in a different population, reported evidence of online positive psychology interventions on healthy adults. ‘Happify Health’, a digital psychological well‐being tool, was tested in a pilot RCT for loneliness and found that in‐built exercises like mindfulness and gratitude allowed them to build their coping skills towards managing isolation (Boucher et al., 2021). However, studies comparing the same type of PPI in in‐person versus virtual settings are required to consider whether and how the mode of delivery affects effectiveness.

Overall, the positive psychology interventions investigated in this review showed evidence in improving subjective well‐being, physical health symptoms like nausea and labour pain, and depression, although not all pregnant women gained significant benefits. This shows that different characteristics of the intervention, such as the specific theories on which the interventions were based, the various positive psychology components highlighted within all PPIs, duration, delivery format and differing beneficial focus between mental and physical well‐being, influence the effectiveness of these interventions for pregnant women.

### Limitations and future directions

As a first review of PPI during pregnancy, the findings should be considered with limitations. Based on quality assessment tools, selected literature had methodological insufficient reporting, such as unreported age and fidelity of psychometrics tools used to examine the effects on outcomes (Samavi et al., 2018), programme differentiation in O'Leary (2015) for not tracking completion rates for their mindfulness intervention, lack of qualitative follow‐up for a pilot study (Barber & Masters‐Awatere, 2022) and adherence rates (Matvienko‐Sikar & Dockray, 2017). Considering the diversity in outcomes, the focus of PPIs ranged from hope‐based, meaning‐making, gratitude‐based interventions, for example, and different phases of the pregnancy and postpartum journey, generalising the intervention effects were not possible. As a result, the findings of this review should be interpreted as understanding the general effects and influences of PPIs during pregnancy more than observing the differences between individual intervention types. Similarly, length of intervention varied greatly in the selected list of studies from single to multi‐session interventions. Hence, providing specific recommendations on optimal PPI duration is not possible at this stage, as the observations remain varied and speculative in the absence of controlled comparison in a few selected studies. Our systematic review only included English language studies, which meant positive psychology research such as of Mostafaei et al. (2020) had to be excluded. Future reviews could consider alternative methods such as collaborative efforts with researchers across countries to include studies from diverse languages, permitting the opportunity to make cross‐cultural comparisons on the use and effectiveness of PPI during pregnancy. Lastly, given the known complexities of conducting a systematic review, the possibility of imprecise search strategies is not disqualified; therefore, some studies might have been missed by chance.

## AUTHOR CONTRIBUTIONS

**Tharunnia M.S Ganesan**: Study conceptualisation, PRISMA registration, conducting systematic literature review, writing – original manuscript and writing – review and editing. **Zuhrah Beevi**: Study conceptualisation, funding acquisition, second reviewer of systematic literature review, and review and editing of the manuscript. **Alan J. Gow**: Supervision, study conceptualisation, and review and editing of the manuscript. **Guek Nee Ke**: Supervision and review and editing of the manuscript.

## CONFLICT OF INTEREST STATEMENT

The authors declare that there are no relevant financial or nonfinancial interests to disclose.

## ETHICS STATEMENT

The authors have nothing to report.

## ADDITIONAL INFORMATION

In two instances, authors were contacted to clarify study details. Samavi et al. (2018) had not reported the age of the pregnant women included in their hope therapy study. Although no response was received, this study was, however, included because there was no indication that the population was underaged. Yue (2022) was excluded from this review as more information regarding their intervention was required at the screening stage itself, but no response was received after further information was requested.

## Data Availability

Data sharing is not applicable as the data are drawn from already published literature, which can be obtained from the references.
